# Prevalence of poor glycemic control among elderly type 2 diabetes patients in rural India

**DOI:** 10.6026/973206300220630

**Published:** 2026-02-28

**Authors:** Malarkodi M, Aruna S, Jeevithan S, Sudha M

**Affiliations:** 1 Department of Community Health Nursing, KMCH College of Nursing, Coimbatore, The Tamil Nadu DR. M.G.R Medical University, Chennai, India; 2 Department of Community health Nursing, Sri Ramachandra Institute of Higher Education and Research (Deemed to be University), Porur, Chennai, Tamil Nadu, India; 3 Department of Community Medicine, KMCH Institute of Health Sciences and Research, Coimbatore, The Tamil Nadu DR. M.G.R Medical University, Chennai, India; 4 Department of General Medicine, Sri Ramachandra Medical College & Research Institute, Sri Ramachandra Institute of Higher Education and Research (DU), Porur, Chennai, Tamil Nadu, India

**Keywords:** Poor glycemic control, type 2diabetes, elderly, HbA1c, rural health

## Abstract

Poor glycemic control remains a major public health problem among elderly patients with type 2 diabetes in rural India. Therefore, it is of interest to assess poor glycemic control among 378 elderly patients with type 2 diabetes attending a rural health centre in Coimbatore. Data were collected using structured interviews and HbA1c measurements to assess glycemic status. Data show that 77.5% of participants had inadequate glycemic control with HbA1c levels above 7. A significant association was observed between longer duration of diabetes and poor glycemic control, while no significant associations were found with age, gender, education, treatment type, comorbidities, or health education. This study advances knowledge by highlighting a high prevalence of poor glycemic control among elderly patients with type 2 diabetes in a rural setting. It identifies longer duration of diabetes as a key determinant, emphasizing the need for duration-focused glycemic management.

## Background:

Type 2 diabetes mellitus (T2DM) is a major public health challenge worldwide and particularly in India, where it is estimated that more than 77 million individuals are living with diabetes. The burden is growing rapidly in rural areas due to demographic transitions, urbanization of rural lifestyles and changes in diet and physical activity patterns [1]. Among the elderly, the prevalence of T2DM is increasing and poor glycemic control (HbA1c > 7%) contributes significantly to complications such as cardiovascular disease, neuropathy, retinopathy and nephropathy [2]. In rural Indian settings, poor healthcare infrastructure, limited access to regular screening and monitoring, low literacy rates and poor awareness about diabetes management are key challenges that exacerbate poor glycemic control [3]. Studies from various parts of India consistently report a high prevalence of poor glycemic control among T2DM patients, ranging from approximately 50% to nearly 70% [4, 5]. For instance, a recent study in Maharashtra highlighted the role of gender-specific factors and socioeconomic determinants in influencing glycemic control, emphasizing the need for tailored interventions [1]. Another study from Mysuru district reported poor medication adherence, inadequate dietary compliance and infrequent check-ups as key drivers of poor glycemic control [2]. In Kerala, poor lifestyle modification and lack of physical activity have been linked to high rates of uncontrolled diabetes among rural patients [6]. Therefore, it is of interest to determine the prevalence of poor glycemic control among elderly T2DM patients at KMCH RHTC, identify associated sociodemographic and clinical factors and evaluate their relationship with diabetes duration and other variables.

## Methodology:

## Study design and setting:

A descriptive cross-sectional study was conducted at KMCH Rural Health Training Centre (RHTC), Coimbatore, from January 10-16, 2025, targeting elderly patients with type 2 diabetes mellitus.

## Population and sampling:

The accessible population included T2DM patients receiving treatment at KMCH RHTC. A sample of 378 patients was selected using simple random sampling, calculated based on a sample size of 358 plus 20 additional samples to account for non-response. Inclusion criteria were patients aged 60-75 years, diagnosed with T2DM for >5 years and of both genders. Patients with complications were excluded.

## Data collection:

Data were collected through structured face-to-face interviews (approximately 20 minutes per participant) using a questionnaire covering age, gender, education, occupation, family income, food habits and clinical history (duration of diabetes, treatment type, treatment duration, random blood sugar and HbA1c levels). Glycemic control was assessed using HbA1c, with levels >7 indicating inadequate control. Data analysis was performed using SPSS version 22.

## Data analysis:

Descriptive statistics (frequency, percentage) analyzed demographic and clinical characteristics. Chi-square tests assessed associations between glycemic control and variables (p<0.05). Odds ratios (OR) with 95% confidence intervals (CI) were calculated for significant associations.

## Ethical considerations:

Ethical clearance was obtained from KMCH and SRIHER, with the study registered in the Clinical Trial Registry of India (CTRI). Informed consent was secured from participants, ensuring confidentiality and voluntary participation.

## Results:

Among 378 participants, demographic and clinical characteristics were as follows: Table 1 summarizes that the majority of participants were aged 60-70 years (78%), predominantly male (64%), with primary education (51.9%). Most had diabetes for more than 10 years (62%), used oral hypoglycemic agents alone (55.8%) and 45.2% reported comorbidities. Socioeconomically, 42.1% belonged to the middle class and 37% to the lower middle class. Table 2 shows that a significant association was found between diabetes duration >10 years and poor glycemic control (p=0.03, OR=0.54, 95% CI: 0.31-0.95). No significant associations were observed between glycemic control and age (p=0.42), gender (p=0.13), education (p=0.26), treatment type (p=0.11), comorbidities (p=0.80), or health education (p=0.88), suggesting disease duration as the key predictor of poor control in this elderly rural population.

## Discussion:

This study found a high prevalence of poor glycemic control (77.5%) among elderly type 2 diabetes patients at KMCH RHTC, consistent with reports of 50-80% prevalence in rural India [4]. The significant association between diabetes duration >10 years and poor glycemic control (p=0.03, OR=0.54, 95% CI: 0.31-0.95) aligns with evidence that prolonged disease duration exacerbates glycemic control due to progressive beta-cell dysfunction [7]. For example, a rural study in the Vindhya region found that patients with >10 years of diabetes had significantly higher poor control rates [3]. Another rural study demonstrated a positive association between longer diabetes duration and elevated inflammatory markers (NLR and PLR) and HbA1c levels, supporting this association [8]. The lack of association with age (p=0.42) contrasts with evidence from a North Indian study that reported advancing age (>63 years) as an independent predictor of poor glycemic control and cognitive impairment [9]. Gender showed no significant effect (p=0.13), consistent with findings from a study in Maharashtra where no gender difference was observed after adjusting for sociodemographic variables [1]. Education level did not significantly influence glycemic control (p=0.26), unlike a study from Punjab that found a significant association between lifestyle modification, education level and improved glycemic outcomes [6]. The non-significant effect of health education (p=0.88) suggests current interventions may be inadequate, as a field study in Mysuru demonstrated significant improvements in fasting blood sugar and BMI after structured educational interventions [10]. Treatment type (OHA vs. OHA + lifestyle) showed no significant difference (p=0.11), possibly due to poor adherence to lifestyle changes, as reported in a Karnataka study, which noted that despite satisfactory medication adherence, lifestyle modifications were not adequately followed [11]. Comorbidities had no significant impact (p=0.80), contrasting with evidence showing hypertension and diabetes frequently co-occur in rural elderly, complicating disease management and necessitating integrated care [12]. Socioeconomic status, with 42.1% of patients in the middle class, may influence access to diabetes care. A study in Karnataka highlighted that lower socioeconomic groups faced significant financial barriers and poor accessibility to healthcare, which strongly affected medication adherence among elderly rural patients with T2DM and hypertension [13]. The overall prevalence of poor glycemic control observed aligns with a national analysis that emphasized the role of socio-demographic factors (education and income) in influencing treatment utilization patterns among elderly diabetics in India [14]. Longer diabetes duration was a key predictor of poor control, corroborated by a Vindhya region study where 68.8% of rural diabetics had poor glycemic control and disease duration was a significant factor [3]. Medication adherence remains a critical challenge. A rural Eastern India study found non-adherence rates at approximately 33% for diabetes, primarily due to medication costs, adverse events and declining memory in elderly patients [15]. Lifestyle modifications also play a significant role. Research from rural Punjab reported that poor adherence to lifestyle changes was prevalent among elderly diabetics and significantly associated with higher random blood sugar and diabetes-related complications [6]. Comorbid conditions did not significantly affect glycemic control in this study, but broader evidence shows that T2DM patients with comorbidities such as hypertension and dyslipidemia require more integrated care and frequently fail to meet guideline targets [16]. Clinician perspectives further underscore the importance of affordable, individualized care. The DESERVE India expert consensus highlighted the widespread difficulties clinicians face in managing rural, resource-challenged diabetics and emphasized low-cost therapies to improve adherence [17]. These findings support a pressing need for tailored, community-based interventions that address financial, educational and behavioral determinants of diabetes care.

## Conclusion:

We show that poor glycemic control is highly prevalent among elderly patients with type 2 diabetes in a rural healthcare setting. Longer duration of diabetes emerged as the key determinant of inadequate glycemic control. Other demographic and clinical factors showed no significant association with glycemic status. Thus, we show the need for duration-focused, community-based and adherence-oriented interventions to improve glycemic outcomes in elderly rural populations.

## Figures and Tables

**Table 1:** Demographic and clinical characteristics of participants (n=378)

**S. No.**	**Variable**	**Category**	**No. of Participants**	**Percentage (%)**
1	Age	60-65 years	154	40.7
		66-70 years	141	37.3
		71-75 years	83	22
2	Gender	Male	242	64
		Female	136	36
3	Education	Illiterate	64	16.9
		Primary	196	51.9
		Secondary	92	24.3
		Graduate	26	6.9
4	Duration of Diabetes	5-10 years	144	38.1
		11-15 years	158	41.8
		>15 years	76	20.1
5	Treatment Type	OHA	211	55.8
		OHA + Lifestyle	167	44.2
6	Comorbidities	Yes	171	45.2
		No	207	54.8
7	Socioeconomic Status (Modified B.G. Prasad)	Middle class	159	42.1
		Lower middle class	140	37
		Lower class	50	13.2
		Upper middle class	26	6.9
		Upper class	3	0.8

**Table 2:** Association of demographic and clinical factors with glycemic control

**Variables**	**Adequate Glycemic Control (HbA1c<7)**	**Inadequate Glycemic Control (HbA1c>7)**	P-value*
**Age**			
≤70 years	69 (81.2%)	226 (77.1%)	0.42
>70 years	16 (18.8%)	67 (22.9%)	
**Gender**			
Males	60 (71.4%)	182 (62.5%)	0.13
Females	24 (28.6%)	109 (37.5%)	
**Education**			
Illiterate	11 (12.9%)	53 (18.1%)	0.26
Literate	74 (87.1%)	240 (81.9%)	
**Duration of Diabetes**			
>10 years	61 (71.8%)	241 (82.3%)	0.03
≤10 years	24 (28.2%)	52 (17.7%)	
**Type of Treatment**			
OHA	41 (48.2%)	170 (58.0%)	0.11
OHA + Lifestyle	44 (51.8%)	123 (42.0%)	
**Comorbidities**			
Yes	37 (43.5%)	134 (45.7%)	0.8
No	48 (56.5%)	159 (54.3%)	
**Health Education**			
Yes	48 (56.5%)	168 (57.3%)	0.88
No	37 (43.5%)	125 (42.7%)	
*Chi-square test;
OHA-Oral Hypoglycemic Agents
